# Population dynamics and genome-wide selection scan for dogs in Chernobyl

**DOI:** 10.1186/s40575-023-00124-1

**Published:** 2023-03-08

**Authors:** Megan N. Dillon, Rachael Thomas, Timothy A. Mousseau, Jennifer A. Betz, Norman J. Kleiman, Martha O. Burford Reiskind, Matthew Breen

**Affiliations:** 1 Department of Molecular Biomedical Sciences, College of Veterinary Medicine, North Carolina State University, Raleigh, NC USA; 2 Department of Biological Sciences, North Carolina State University, Raleigh, NC USA; 3 Department of Biological Sciences, University of South Carolina, Columbia, SC USA; 4 Visiting Veterinarians International, 9825 SE Tower Dr, Damascus, OR USA; 5 Department of Environmental Health Sciences, Mailman School of Public Health, Columbia University, New York, NY USA; 6 Comparative Medicine Institute, North Carolina State University, Raleigh, NC USA; 7 Center for Human Health and the Environment, North Carolina State University, Raleigh, NC USA; 8 Cancer Genetics, UNC Lineberger Comprehensive Cancer Center, University of North Carolina, Chapel Hill, NC USA; 9 Duke Cancer Institute, Duke University, Durham, NC USA

**Keywords:** Outlier analysis, Population structure, Environmental contamination, Chernobyl dogs

## Abstract

**Background:**

Natural and anthropogenic disasters can have long-lasting impacts on the genetics and structure of impacted populations. The 1986 Chernobyl Nuclear Power Plant disaster led to extensive contamination of the local environment and the wildlife therein. Several ecological, environmental, and genetic studies reported various effects of this disaster on animal, insect, and plant species; however, little work has been done to investigate the genetics of the free-breeding dogs that occupy the Chernobyl Exclusion Zone (CEZ).

**Results:**

We define the population genetic structure of two groups of dogs that reside within the CEZ, one around the reactor site itself and another living within Chernobyl City. We found little evidence of gene flow and a significant degree of genetic differentiation between the two populations dogs, suggesting that these are two distinct populations despite occupying areas located just 16 km apart. With an *F*_*ST*_-based outlier analysis, we then performed a genome-wide scan for evidence of directional selection within the dog populations. We found 391 outlier loci associated with genomic regions influenced by directional selection, from which we identified 52 candidate genes.

**Conclusions:**

Our genome scan highlighted outlier loci within or near genomic regions under directional selection, possibly in response to the multi-generational exposure faced. In defining the population structure and identifying candidate genes for these dog populations, we take steps towards understanding how these types of prolonged exposures have impacted these populations.

**Supplementary Information:**

The online version contains supplementary material available at 10.1186/s40575-023-00124-1.

## Abstract (summary)

Wildlife populations can be greatly affected by disasters, whether they are natural or man-made. Disasters that result in contamination or habitat destruction can result in population declines or influence wildlife adaptation to these adverse environmental changes. The Chernobyl nuclear power plant disaster released an enormous quantity of ionizing radiation into the surrounding environment. Abandonment of military and industrial facilities, as well as subsequent cleanup and remediation efforts, resulted in further environmental contamination by a variety of non-radioactive toxic metals, chemicals, and compounds. Earlier studies investigated local wildlife responses to some of these exposures. In this study, we address the impact of this disaster on the population structure of free-breeding dogs that live around the power plant and in the nearby city of Chernobyl. In particular, we use genetic approaches to understand how these two populations of dogs interact and their breed composition, so that we may begin to understand how these populations have adapted to over 30 years of exposure to this harsh environment. In this foundational study we determined that while the two local populations of dogs are separated by only 16 km, they have very low rates of interpopulation migration. We also detected genetic evidence that suggests that these population may have adapted to exposures faced over many generations. In future studies, we aim to determine if the genetic variation detected is indeed a biological response to enable survival after multi-generational exposures to radiation, heavy metals, organic toxins, or other environmental contaminants. In this way, we then understand how the impact of environmental catastrophes such as the Chernobyl nuclear disaster can influence animal populations.

**Supplementary Information:**

The online version contains supplementary material available at 10.1186/s40575-023-00124-1.

## Background

Large-scale disasters, both natural and anthropogenic, can have multiple direct and indirect impacts on wildlife populations. Natural disasters such as tornadoes, volcanic eruptions, and hurricanes, as well as man-made disasters such as oil spills, chemical leaks, and nuclear accidents can have detrimental impacts on local species, lasting for months or even years after the incident [1–4]. Environmental contamination, habitat destruction, and other stressors brought on by disasters can shape the local wildlife populations quickly, especially when the ecological changes can be extreme. These stressors can prompt changes in the genetic population structure, including loss of genetic diversity or selective sweeps. For example, drastic changes such as pollution of the local environment can induce rapid evolution, as evidenced by adaptive toxicant resistance in Gulf killifish [5]. Carcinogenic contaminants can also have direct impacts on the DNA of individuals in the population, such as inducing deleterious point mutations or chromosomal rearrangements [6, 7].

One such disaster with lasting effects was the nuclear disaster in the former Soviet Union at the Chernobyl Nuclear Power Plant (CNPP) on April 26, 1986. This catastrophe resulted in the release of over 5,000 petabecquerels (PBq) of radioisotopes, including 134-Iodine, 90-Strontium and 137-Cesium, into the surrounding environment [8]. Within 48 h of the accident, tens of thousands of residents from nearby towns and villages were evacuated. An area of approximately 700 km^2^ (~ 30 km radius from the power plant) that spans regions of northeastern Ukraine and southern Belarus was established as the Chernobyl Exclusion Zone (CEZ). Heavy metals, organics, and other environmental toxins left behind following decontamination and remediation efforts, along with the abandonment of military bases and industrial complexes, contributes to ongoing contamination of the locale [9–12]. About 150,000 km^2^ of land throughout Ukraine, Belarus, and Russia, and especially in the area surrounding the now decommissioned reactors of the CNPP, remains contaminated and predominantly abandoned, other than a few remaining elderly residents [13]. Though 36 years have passed, the roughly 30-year half-life of the predominant radionuclides, 137-Cs and 90-Sr, means that the threat posed by radioactive contamination will continue for another century. Some areas, especially regions of the former “Red Forest”, remain significantly more contaminated than others. In addition, spontaneous periodic wildfires near the power plant contribute to resuspension of radionuclides and these events are likely to continue [14]. The Russian army occupation of the CNPP in Spring 2022 also led to soil disturbances and a reported increase in radioactivity from around 1 millisievert (mSv) per year to 6.5 mSv/yr in the immediate vicinity of the CNPP, although this increase does not warrant a threat to public health according to a recent assessment by the International Atomic Energy Agency (IAEA) assessment [13, 15]. The potential of natural disasters and anthropogenic disturbances to increase the local radioactivity raises concern about our understanding of how these events continue to impact wildlife and cause adverse environmental impacts.

The Belarusian side of the CEZ was established as the Polesie State Radioecological Preserve and includes a formerly less populated, marshy environment, while the Ukrainian side remains as an unofficial wildlife refuge as it is largely uninhabited by humans. Nevertheless, an established animal presence remains, and in some cases, due to the absence of human activity, may be thriving. Several studies have investigated the impact of persistent radiation exposure, as well as other contaminants, on the wildlife in the CEZ. However, there remains a lack of consensus on how this toxic environment has impacted various species. Ecological studies within the CEZ highlight a reduced abundance of mammals in areas of higher radioactive contamination and research on dose reconstruction supports this reduction [16, 17]. In contrast, other studies found no discernible effects on abundance, even in heavily contaminated regions [18, 19]. Recent work has highlighted local adaptation in different species, including an adaptive response to oxidative stress in bird species [20]. Hancock et al. [21] reportedly found evidence of genetic response and adaptation to historic dose radiation rates at the trans-generational level in voles. Despite these findings, few studies consider the combined effects of chemical mixtures that include ionizing radiation, heavy metals, organics, pesticides, and other toxicants present within the CEZ.

Though Chernobyl wildlife has been the subject of previous ecological and genetic studies (e.g., [22–24]), little is known about the genetics of a population of over 500 dogs occupying the area surrounding the CNPP and Chernobyl City. This dog population has expanded in the decades following the accident and is thought to be comprised at least partially of descendants of pets left behind during the chaotic evacuation in 1986 [25]. In this scenario, it is intriguing to understand to what extent the descendants of these abandoned dogs have adapted to survive and sustain a growing population under these extreme environmental conditions. Several prior studies have demonstrated that the dog may be regarded as a sentinel species for the resultant health impacts of a wide range of human environmental exposures [26, 27]. Understanding and extending the genetic and health impacts of the exposure to both radiological and chemical insults in these dogs will strengthen the broader understanding of how these types of adverse environmental stressors can impact human health. This observation is made all the more relevant by the construction and operation of two new interim storage facilities (ISF), designed to process and store nuclear waste within the CEZ, ISF1 and ISF2 [28]. In addition to the hundreds of workers employed to construct, maintain, and operate these facilities, many hundreds of other workers commute daily by train from the nearby city of Slavutych to continue work on remediation, deconstruction, and maintenance of the former CNPP. These thousands of daily workers are exposed to many of the same potential environmental hazards in soil, water, and air as the resident dog populations, and thus examination of adverse health effects in these animals may serve as proxy for a variety of human exposures.

**Fig. 1:**
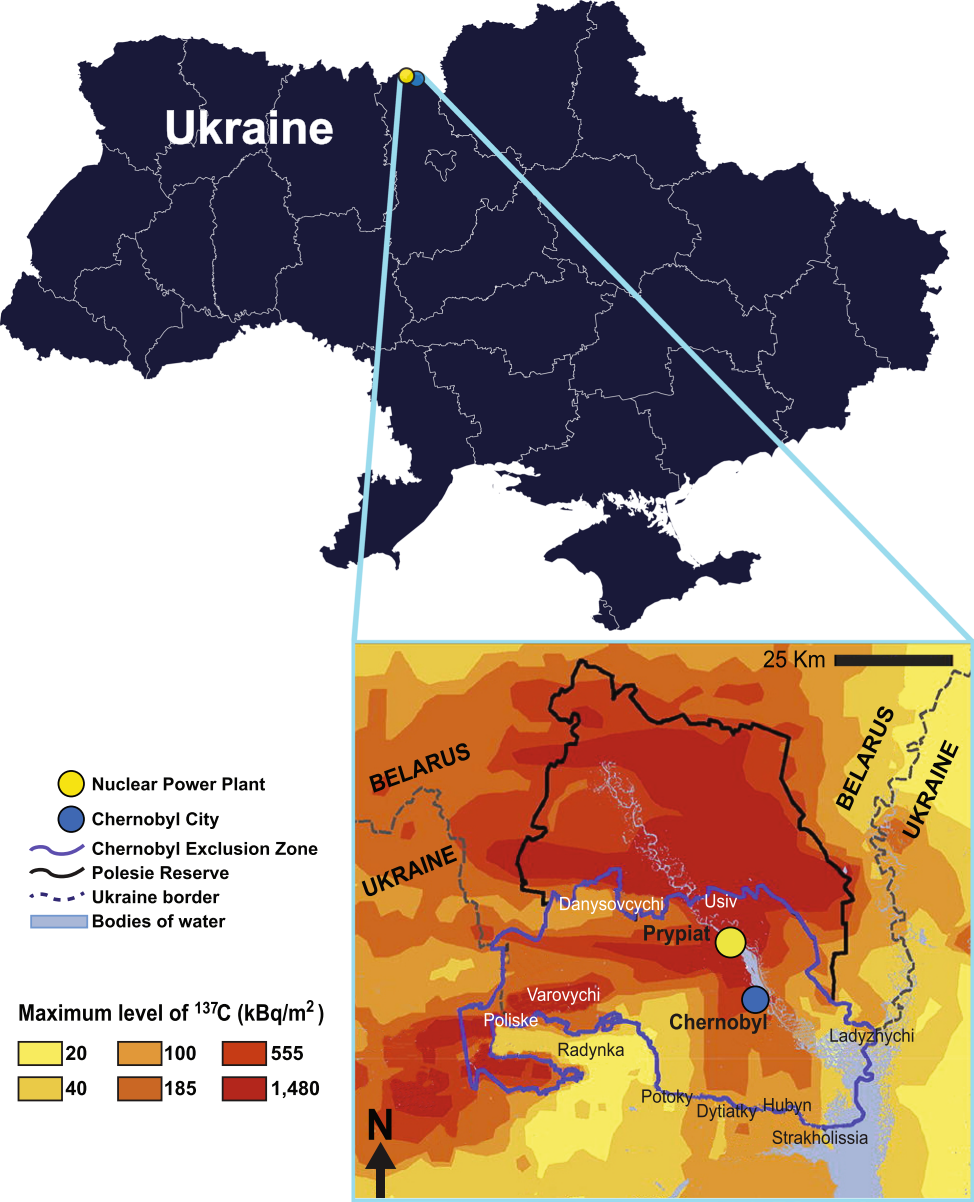
Map of sampling locations. Zoomed in portion of map highlights cesium deposition across Chernobyl Exclusion Zone (blue outline) in Northern Ukraine and a portion of Southern Belarus, adapted from Ager et al. [28]. Sampling locations are indicated, with Nuclear Power Plant (*N* = 60) in yellow and Chernobyl City (*N* = 56) in blue

Our goal in this study was to 1) understand the genetic structure of these dog populations and the degree of isolation that exists within them, and 2) scan their genomes for signatures of directional selection possibly tied to local adaptation within the contaminated area. We addressed these goals through genetic analyses of two populations of free-breeding dogs within the CEZ that face different degrees of exposure ([29]; Fig. 1): dogs sampled in close proximity to the Chernobyl Nuclear Power Plant (hereafter, Nuclear Power Plant dog population) and dogs sampled approximately 16.5 km away from the CNPP in Chernobyl City (Chernobyl City dog population). By establishing the population structure and relatedness between these populations, we can better tease out any genomic differences related to directional selection. This will then help us to assess the prolonged exposure of these contaminants for dog populations. These dogs present a unique opportunity to study the genomic response to multi-generational exposure to environmental hazards.

## Methods

### Sample collection and genomic DNA isolation

Blood samples were collected from semi-feral dogs captured around the Chernobyl Nuclear Power Plant (NPP) and 16.5 km away in Chernobyl City (CC). Blood samples were taken during sterilization and vaccination procedures conducted by the Clean Futures Fund Dogs of Chernobyl program in 2018 and 2019. In total, 116 unique dogs were included in the study, 60 from Nuclear Power Plant and 56 from Chernobyl City (Fig. 1). Blood samples were preserved in PAXGene Blood DNA tubes (Qiagen) and transported at 0 °C back to the United States for DNA extraction. We isolated genomic DNA from 500 μL of whole blood using the Maxwell RSC Whole Blood DNA kit (Promega) according to the manufacturer’s protocol. Following conventional QC for integrity and concentration, each DNA sample was genotyped using the Axiom Canine HD array (Thermo Fisher Scientific), yielding data for over 710,000 single nucleotide polymorphism (SNP) loci aligned to the CanFam3 genome build. All animal protocols, handling, husbandry, and care were approved by the Columbia University Institutional Animal Care and Use Committees (IACUC).

### Variant filtering

We filtered variants through PLINK v1.9 [30] for all 116 unique individuals, and loci with a minor allele frequency less than 5% (–maf 0.05) or that were missing genotype calls in any individual (–geno 0) were removed. We used the resultant 301,023 SNPs (hereafter referred to as the 301 K Set) for investigating genetic differentiation, population assignment, and inbreeding within this study. To perform a stringent outlier analysis, we also generated a reduced dataset (84 K set) by applying a minor allele frequency threshold of 35% to the 301 K set. For analyzing runs of homozygosity (ROH) across the genome, we removed loci with missing genotypes (–geno 0) and selected only autosomal SNPs (–chr 1:38). The ROH dataset comprised 427,455 (427 K set).

### Population assignment and clustering analysis

We conducted a principal components analysis (PCA) and a discriminant analysis of principal components (DAPC) using the 301 K Set in ADEGENET [31] (functions: glPca, dapc) to investigate genetic differentiation between the two populations. We retained 30 principal components (PCs) following cross-validation procedures in ADEGENET (xvalDapc). Clustering procedures for the initial DAPC highlighted 12 individuals that grouped with the opposite capture location (function: find.clusters). We removed these individuals before further analysis and used SNP data for the remaining 104 dogs for calculating *F*_*ST*_, measuring heterozygosity, and identifying outlier loci.

### Genetic differentiation and inbreeding

We calculated pairwise *F*_*ST*_ using Weir and Cockerham’s estimator [32] over 1,000 bootstraps through the hierfstat package for R [33]. Due to the relative nature of *F*_*ST*_ estimates, we conducted a second analysis to compare our pairwise *F*_*ST*_ estimate to the pairwise values between other free-breeding dog populations. For this second analysis, we used open-source SNP data acquired and published by Pilot et al. ([34, 35]; hereafter, Pilot et al. dataset) and used hierfstat to also calculate pairwise *F*_*ST*_ amongst free-breeding dog populations across Europe and Asia. Pilot et al. [34] genotyped 324 individuals with the CanineHD Whole-Genome Genotyping BeadChip (Illumina), so, to maintain consistency in our comparisons, we filtered each SNP dataset to retain only those SNPs included on both the Illumina and Affymetrix arrays and which were present in at least 50% of individuals in both datasets. This filtering resulted in 147,592 SNPs (147 K set). We recalculated pairwise *F*_*ST*_ for Chernobyl City and Nuclear Power Plant populations and then calculated pairwise *F*_*ST*_ for the free-breeding dog populations included in the Pilot et al. dataset, which included dogs sampled in Poland, Slovenia, Iraq, Saudi Arabia, Armenia, Central Russia, Eastern Russia, Kazakhstan, Tajikistan, China, Mongolia, and Thailand.

We used the hierfstat package to measure observed and expected heterozygosity for each locus, which we compared across populations with a paired t-test with the alpha corrected for multiple comparisons (corrected α = α / 301,023). We used the R package SNPRelate [36] to calculate individual inbreeding coefficients with Visscher’s estimator as described by Yang et al. [37] and compared measures between populations through Welch’s two sample t-test. As an additional measure of inbreeding, we analyzed runs of homozygosity (ROH) per individual with PLINK (–homozyg). For this, we utilized the 427 K Set for all 116 unique individuals, subset for each population, because minor allele frequency filtering can overlook homozygous stretches which limits ROH detection [38]. This also allowed for more comprehensive coverage for the scans. We selected parameters based on Sams and Boyko [39] and Morrill et al. [40] to best accommodate SNP coverage for our data set: ROH longer than 500 kb (–homozyg-kb 500), contained at least 50 SNPs (–homozyg-snp 50), and contained no heterozygous calls (–homozyg-window-het 0). Inverse density (Kb/SNP) and gap size thresholds were set high (–homozyg-density 5000, –homozyg-gap 1000) to ignore [39]. We then calculated each individual’s *F*_ROH_, using the proportion of genome covered by ROH as a measure of inbreeding [38]. The *F*_ROH_ scores, averaged across populations, were compared using Welch’s Two Sample t-test.

### Migration and gene flow

To generate a measure of gene flow between the populations, we conducted analyses with MIGRATE using Bayesian inference [41–43]. Beginning with the 301 K Set for the 116 unique individuals, we used PLINK to filter out alleles with a minor allele frequency under 50% (–maf 0.5) to target the most variable sites [44]. We evaluated four different migration models for the 1,143 resultant loci: a full migration model, two unidirectional migration models, and a panmixia model. For each locus, the program visited 10,000,000 steps per parameter (a*b*c) following a burn-in of 1,000,000 steps. We utilized the default static heating scheme of four chains with a Bayesian prior range of 0–0.01 for Θ and a range of 0–1,000,000 for M. We calculated log marginal likelihoods (lmL) based on the Bezier approximation scores to select the model that best described the migration patterns [45]. MIGRATE generates Θ values per population, representing the product of effective population size and mutation rate (4Ne*mu), and M, a mutation rate scaled migration estimation (Migration/mu). These measures can then be used to estimate how many migrants there are per four generations, and how these contribute to the genetic diversity of a population relative to mutation rate.

### Breed analysis

We submitted blood-derived DNA from each dog for analysis on the Wisdom Panel™ (MARS). Using the calculated percentages of breed matches, we classified predominant breeds as a breed contributing more than 10% to the genetic lineage for each dog. We compared how many breeds were present in each dog and analyzed the breed counts between the populations using an unpaired student’s t-test. We also utilized a PCA for breed composition between the populations. We compared the two populations for the number of predominant breeds per individual and the maximum percentage of a single breed found within an individual. We also looked for significant correlations between these breed-associated quantifications and ROH measures through calculation of Pearson’s correlation coefficient in the stats R package (functions: cor, cor_pmat).

### Outlier analysis

We used LOSITAN to identify outlier loci associated with directional selection [46]. Outlier analyses such as these frequently produce some degree of false positives [47]. To overcome this, we selected the 84 K set with a higher MAF threshold for this analysis to highlight the strongest signals of selection and biasing towards genomic regions with large effects. We included only the 104 dogs that grouped with their capture location in the DAPC for better identification of the differentiating loci in each population. We ran 1,000,000 simulations and applied a 95% confidence interval and a false discovery rate of 0.1. We considered loci significant when designated as ‘candidate positive selection’ and when the calculated *F*_*ST*_ was higher than the expected *F*_*ST*_ in all simulations (P(Simulated *F*_*ST*_ < sample *F*_*ST*_) = 1).

The genome location of each significant SNP identified using LOSITAN was expanded to a 10 kb genomic interval of CanFam3 (5 kb either side of the SNP coordinates). We then surveyed each of the 10 kb regions for the presence of genes and identified corresponding gene ontology (GO) terms through the Mouse Genome Informatics Batch Query Search [48]. GO terms were evaluated for putative associations towards the exposures faced within the environment (e.g., GO:0,010,212 “response to ionizing radiation”).

## Results

### Population assignment and clustering analysis

**Fig. 2:**
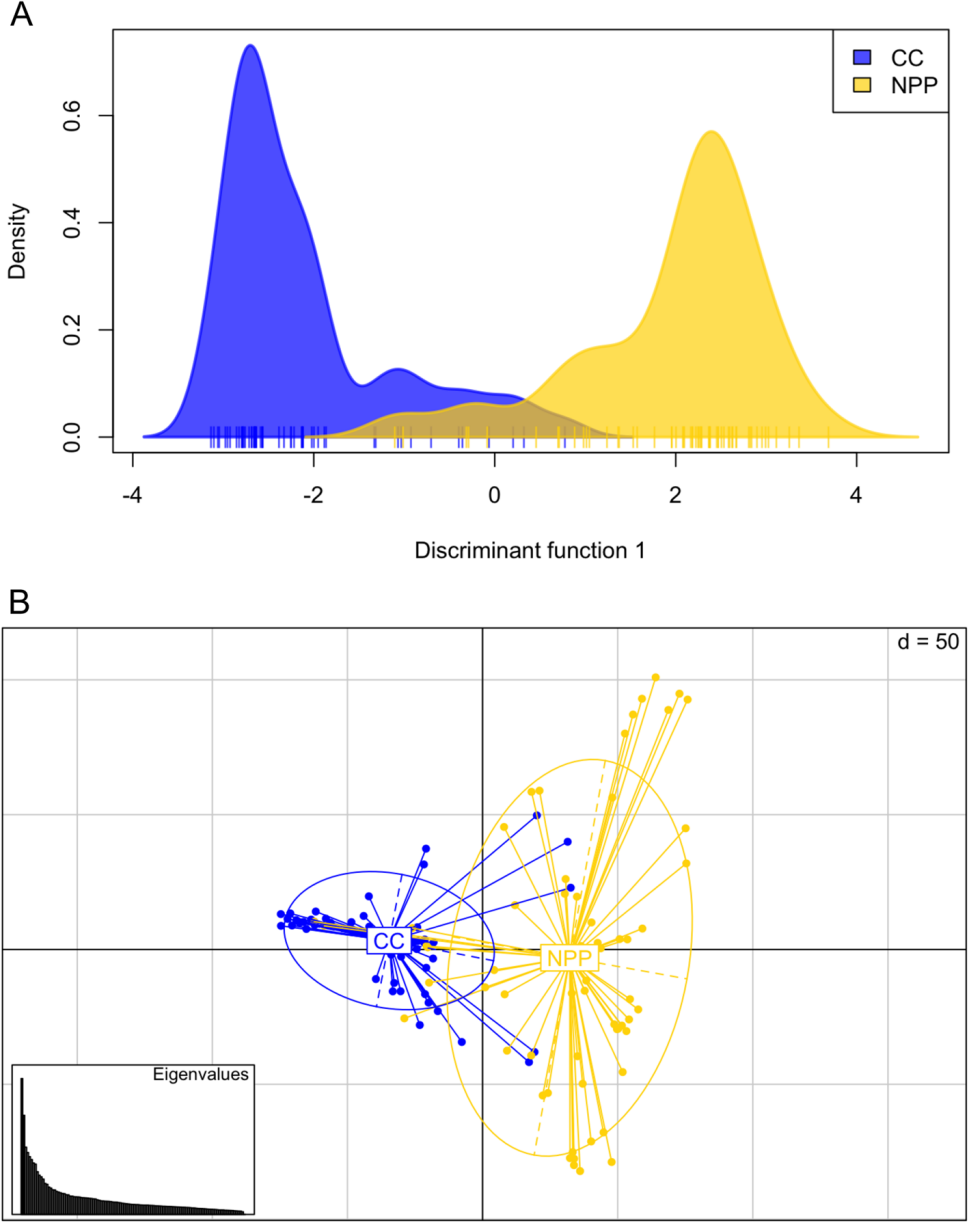
DAPC and PCA highlight population clustering using SNP data (301 K set). **A** Discriminant analysis of principal components highlights separation between populations, plotted across discriminant function 1. We retained 30 principal components and one discriminant axis after cross-validation procedures. **B** Plot of principal component analysis for same SNP data with 30 retained principal components. PC1 (7.2% variance explained) is plotted along the x-axis and PC2 (5.2% variance explained) is plotted along the y-axis

Both DAPC and PCA showed discrimination between the Nuclear Power Plant and Chernobyl City populations, and clustering highlighted a similar structure (Fig. 2). Most of the individuals clustered with their respective population, but 12 dogs grouped with the opposite location (7 from Nuclear Power Plant, 5 from Chernobyl City). Plotting across the discriminant axis showed very similar trends of population separation with minimal crossover. We removed individuals that grouped with the opposite capture location for the outlier analysis and calculation of *F*_*ST*_ measures to allow for the best estimation of loci driving genetic differentiation.

### Genetic differentiation and inbreeding

The calculation of the pairwise *F*_*ST*_ corroborated the patterns found via PCA and DAPC (*F*_*ST*_ = 0.0574; p < 0.01). For our second *F*_*ST*_ analysis, where we aimed to compare our results to the Eurasian free-breeding dog populations using the 147 K set, we found a similar estimate for our populations (*F*_*ST*_ = 0.0572)_*.*_ The pairwise *F*_*ST*_ measures across free-breeding dog populations within the Pilot et al. dataset ranged from 0.0043 to 0.0889 (Table 1).

**Table 1:** Pairwise* F*_*ST*_ matrix for Pilot et al. (2015) [34] data set, with measures less than that estimated for CC v. NPP (0.0572) bolded and italicized

	**Bulgaria**	**Poland**	**Slovenia**	**Iraq**	**Saudi Arabia 1**	**Saudi Arabia 2**	**Armenia**	**Central Russia**	**Kazakhstan**	**Tajikistan**	**China**	**Mongolia**	**Thailand**
Poland	***0.0095***												
Slovenia	***0.0083***	***0.0073***											
Iraq	0.0692	0.0751	0.0726										
Saudi Arabia 1	0.0635	0.0751	0.0716	0.0744									
Saudi Arabia 2	0.0807	0.0915	0.0881	0.09	***0.0423***								
Armenia	***0.0059***	***0.0112***	***0.01***	***0.0559***	***0.051***	0.0641							
Central Russia	***0.0111***	***0.0123***	***0.0128***	0.0702	0.0686	0.0832	***0.0104***						
Kazakhstan	***0.0052***	***0.0071***	***0.0066***	0.0612	0.0577	0.0724	***0.0039***	***0.0074***					
Tajikistan	***0.007***	***0.0116***	***0.0137***	0.0596	***0.0539***	0.0671	***0.0051***	***0.0094***	***0.0042***				
China	***0.035***	***0.0396***	***0.0364***	0.0786	0.0653	0.0753	***0.0286***	***0.032***	***0.0268***	***0.0256***			
Mongolia	***0.0147***	***0.0199***	***0.0202***	0.0624	***0.0546***	0.0673	***0.0115***	***0.0145***	***0.0094***	***0.0062***	***0.0147***		
Thailand	***0.0358***	***0.0389***	***0.0337***	0.0757	0.0668	0.0739	***0.0299***	***0.035***	***0.0278***	***0.0304***	***0.0105***	***0.0222***	
East Russia	***0.011***	***0.0119***	***0.0127***	0.0729	0.0727	0.0879	***0.0121***	***0.0115***	***0.0083***	***0.0075***	***0.0334***	***0.0146***	***0.0358***

**Fig. 3:**
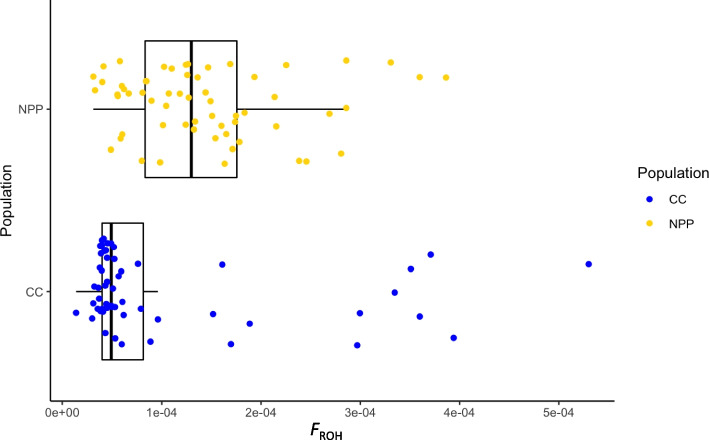
Proportion of the genome covered by runs of homozygosity (FROH) for individuals, plotted by population. Nuclear Power Plant (NPP) had a significantly larger FROH on average than Chernobyl City (CC). Lower and upper hinges of the boxplot indicate 25th and 75th percentiles, and whiskers mark distances of 1.5 * interquartile range from the hinge

Observed and expected heterozygosity per locus were significantly higher within the CC population after Bonferroni corrections for multiple comparisons (*p* < 0.01 for all comparisons; Table 1). Individual inbreeding coefficients were higher on average within the CC population but not significantly different between the populations (*p* = 0.14; Table 2; Supplementary Fig. 1). The Nuclear Power Plant population had a higher quantity of ROH fragments and longer runs on average than the individuals in the Chernobyl City population. Individuals within the Nuclear Power Plant population also had a larger portion of the genome covered with homozygous runs on average than the Chernobyl City population (*p* < 0.05; Table 2, Fig. 3).

**Table 2:** Average heterozygosity and inbreeding calculations across Nuclear Power Plant (NPP) and Chernobyl City (CC) populations. Observed and expected heterozygosity were calculated per locus and averaged across loci and individuals for each population. Paired t-tests were used to test for significant differences between populations, and both measures were found to be significantly different between populations. Individual inbreeding coefficients and proportion of the genome covered by ROH segments (*F*_ROH_) were calculated for each individual and averaged across populations. Welch’s two-sample t-test was used to test for significant differences between populations for these two measures. For resultant *p*-values, * indicates *p* < 0.05; ** indicates *p* < 2.2 × 10^–16^

	Observed Heterozygosity **	**Expected** Heterozygosity **	**Individual Inbreeding Coefficients**	***F***_**ROH**_ *
**NPP**	0.320	0.319	0.003	1.45 × 10^–4^
**CC**	0.337	0.344	0.033	1.02 × 10^–4^

### Migration and gene flow

We found the best fit for these populations with the full, bidirectional migration model based on marginal likelihood estimations (Full Model lmL = 1). We estimated the number of migrants per generation using the modes of Θ and M values for both migration to Chernobyl City from Nuclear Power Plant and to Nuclear Power Plant from Chernobyl City and generated estimates of 0.57 and 0.75 migrants every four generations, respectively (Table 3). Though the parameters are scaled based on mutation rate, which is not estimated here, larger values of M suggest that migration accounts for a larger contribution of genetic diversity than mutation.

**Table 3:** MIGRATE generated modes of posterior distributions for effective population size (Θ) for both Chernobyl City (CC) and Nuclear Power Plant (NPP) and migration (M) for either direction. Θ is the product of effective population size and mutation rate, and M is scaled by mutation rate. Estimates of parameters and 95% credibility intervals based on 10,000,000 visited steps

Parameter	Mode of the Posterior Distribution	95% credibility interval
**Θ**_CC_	0.00090	(0.00089, 0.00092)
**Θ**_NPP_	0.00096	(0.00095, 0.00098)
**M**_CC -> NPP_	833.3	(666.7,1000.0)
**M**_NPP -> CC_	633.3	(400.0, 733.3)

### Breed analysis

**Fig. 4:**
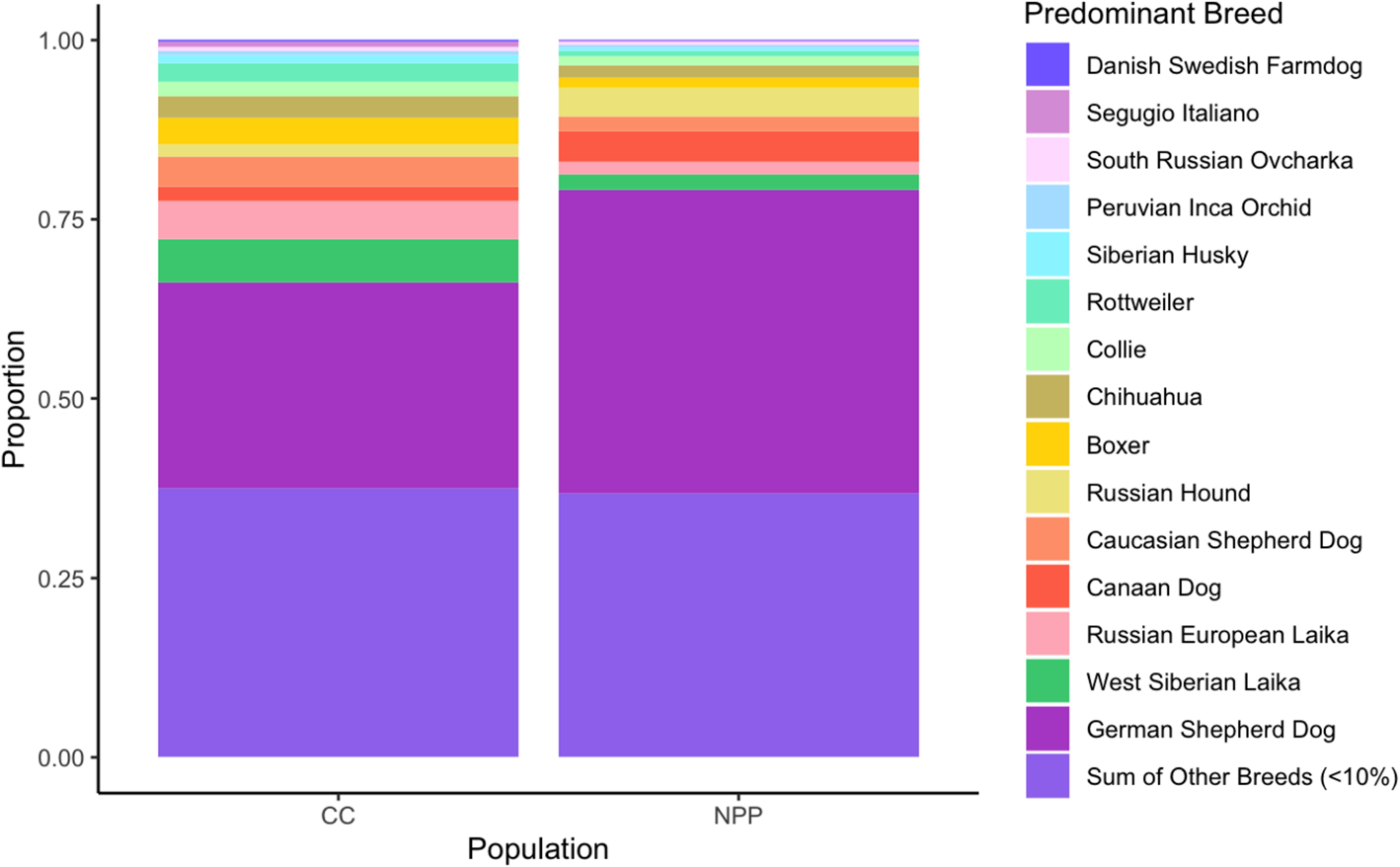
Average breed composition for an individual at Chernobyl City (CC) and Nuclear Power Plant (NPP). SNP data were used to predict breed composition of each dog at CC and NPP, determined by reference to the Wisdom Panel database. Each breed contributing at least 10% to an individual dog is referred to as a predominant breed. Sixteen predominant breeds were identified and all others contributing less than 10% were combined into a single group referred to as ‘Other Breeds’. The stacked histogram reveals that while the populations at CC and NPP shared the same level of contribution by ‘Other Breeds’ there was variation in the contribution of the 16 predominant breeds

While the WISDOM PANEL may not have a comprehensive representation of breeds from which the Nuclear Power Plant and Chernobyl City populations may have been founded, we can still use the approach to compare relative breed composition. All 61 of the Nuclear Power Plant dogs and 52/55 of the Chernobyl City dogs were identified as being at least 10% German Shepherd Dog (GSD), according to the Wisdom Panel™. None of the sampled dogs in either the Nuclear Power Plant or Chernobyl City populations were determined to be purebred, with both populations averaging 25 breed matches per dog. Further investigation of the genetic ancestry of the dogs revealed that 17 of the Nuclear Power Plant dogs and 21 of the Chernobyl City dogs had at least two predominant breeds (defined here as a single breed contributing more than 10%). While GSD was the most observed predominant breed, both populations also had moderate prevalence of Eastern European breeds, such as West Siberian Laika and Caucasian Shepherd Dog (Fig. 4). The Nuclear Power Plant population had a high incidence of Canaan Dog, which was not seen within the Chernobyl City population at the established rate for predominance. The PCA for the breed analysis data did not fully separate the populations, but rather had a large amount of overlap (Supplementary Fig. 2A).

The two populations have a similar breed makeup on average, but dogs from the Nuclear Power Plant population tended to have a higher proportion of a single breed (Supplementary Fig. 2B). To investigate whether a higher proportion of a single breed was tied to higher *F*_ROH_, we compared the breed measures to the different ROH scores. The number of predominant breeds in each dog was not tied to the number of ROH segments, the average size of ROH fragments, nor the *F*_ROH_ for each individual according to Pearson’s correlation coefficient (Supplementary Fig. 3). The maximum breed score of one breed in any individual was also not significantly correlated to the number of ROH segments nor to the *F*_ROH_. There was a weak but significant positive correlation between the maximum breed score and the average size of the ROH segments (Correlation coefficient: 0.18; p-value: 0.049). Since the average size was not a factor considered in evaluation of the* F*_ROH_, we continue to utilize the *F*_ROH_ to evaluate inbreeding.

### Outlier analysis

Through LOSITAN, we found 391 significant outlier loci and identified 165 genes that aligned to CanFam3.1 within 10 Kb of the outlier loci. Fifty-two of these genes, proximal to 67 outlier SNPs, have GO terms that could be associated with exposure to the contamination of the environment at the Nuclear Power Plant. These include GO terms associated with DNA repair, cell cycle signaling, response to radiation, calcium ion binding, and immune function (Supplementary Table 2). One of the candidate genes in proximity to an outlier locus, for example, is X-Ray Repair Cross Complementing 4 (*xrcc4*). *xrcc4* plays a crucial role in repairing ionizing radiation-induced double stranded breaks through non-homologous end-joining in mammals [49]. Contactin Associated Protein 2 (*cntnap2*) is an additional candidate gene and is tied to the inflammatory response [50] through the same pathway (Akt-mTOR) which can be influenced by exposure to radiation [51].

## Discussion

Natural and anthropogenic disasters can cause drastic changes within the environment to which local wildlife populations are sensitive [52]. In this study, we analyzed the population genetic structure of free-breeding dogs living within the Chernobyl Exclusion Zone, an area greatly affected by a well-recognized anthropogenic disaster. Previous studies reported evidence for selection and radio-adaptation for some species living around the Chernobyl Nuclear Power Plant, as summarized in the reviews from Møller and Mousseau [53] and Cannon and Kiang [54]. In classifying the population structure of these dogs, we found putative genomic regions influenced by directional selection and identified candidate genes within the Chernobyl dogs.

We detected a significant degree of genetic differentiation between the two populations of dogs sampled at the Nuclear Power Plant and in Chernobyl City, along with almost complete clustering at the population level through the DAPC and PCA, corroborating trends that were seen in identity by state clustering analyses in Spatola et al. (in press). While the focus of Spatola et al. (in press) was different, the studies are complementary in that while Spatola et al. assessed a larger number of individuals (*n* = 302) each with a smaller number of genotyped loci, we used a much larger number of genotyped loci (~ 3X) and approx. half the number of individuals (*n* = 116) and were able to confirm the population genetic structure they suggested. These results, along with the pairwise *F*_*ST*_ estimates, suggest that the two populations are maintained as separate groups and are breeding predominantly amongst themselves. This was further corroborated by the comparative estimates across different Europasian free-breeding dog populations in the Pilot et al. dataset. Pilot et al. [34], found clustering among free-breeding dog populations of similar areas but with lower degrees of genetic structure across the dataset. Their sampled populations, aside from populations sampled in Middle Eastern countries, had lower pairwise *F*_*ST*_ than Nuclear Power Plant and Chernobyl City, despite having much more geographic distance between them, further suggesting these populations are distinct. We suggest that this high level of differentiation among these two geographically close populations could be prompted by genetic drift, caused by small population sizes and limited migration, or by strong selection not seen in the Eurasian free-breeding dog populations.

Our MIGRATE results support the proposition of genetic differentiation through low estimated migration rate. Despite this, gene flow likely contributes more to generate genetic variation within each population than mutation does because the estimated M value is scaled by mutation rate. Considering this, in conjunction with the results of the MIGRATE analysis, we hypothesize that these two populations likely do not have enough migrants per generation to maintain a well-mixed larger population. These two populations may also likely exist with distinct packs within them, as free-breeding dogs have been shown to form packs similar to those of wild wolves, though they tend towards polygamy more than wolves do [55]. Within these packs, free-breeding dogs may be more likely to maintain the population genetic structure between the two sampled populations. At this stage, however, random genetic drift is also a possible explanation for the differences in allelic frequencies, especially as these populations do not appear to be maintaining a large degree of gene flow. With the more stringent filtering for our outlier analysis, our goal was home in on genomic regions (and thus, genes) more likely to be influenced by stronger, more impactful selection pressures.

We found a slightly reduced degree of expected and observed heterozygosity within the Nuclear Power Plant population, which can be interpreted as a lower degree of genetic diversity for that population. Fuller et al. [56] found that an increased radiation dose rate for aquatic invertebrate populations at Chernobyl was not correlated with reduced genetic diversity – this raises questions about the genetic signature of the founder population which we aim to address in future studies. In addition to finding a different degree of heterozygosity between the Nuclear Power Plant and Chernobyl City populations, we found that the Nuclear Power Plant population had a higher degree of ROH coverage across the genome and larger stretches of homozygous fragments. These longer, more expansive ROHs indicate more recent inbreeding events within the Nuclear Power Plant population. A higher *F*_ROH_ inbreeding score was previously tied to purebred dogs, whereas mixed breed dogs or village dogs had lower proportions of the genome covered by these ROH fragments [40]. To investigate this possible explanation, we considered the ROH measures compared to whether the individual had more than one predominant breed, along with the maximum breed score for each dog. While the Chernobyl City population does have a smaller proportion on average of single breed match and a higher percentage of dogs with two predominant breeds present, we found no significant correlations between these breed measures and the calculated *F*_ROH_. Therefore, the differences between ROH coverage in the populations was not tied to the breed identity of the dogs, but rather can be considered as a representative measure of inbreeding and points towards higher levels of inbreeding within the Nuclear Power Plant population.

Our outlier analysis highlighted almost 400 loci with allele frequencies resembling a response to selection. These loci were proximal to genes that may be under selective pressures and relevant to the varied environmental pressures faced by these dogs. We found that 52 of the genes had associated Gene Ontology (GO) terms for the molecular functions of gene products that are of interest based on a putative response to the exposures from the Chernobyl disaster. These candidate genes were of particular interest because they are involved in functions such as DNA repair and cell cycle checkpoint progression, immune response, and calcium ion binding. Our ongoing studies are designed to generate additional measures to refine the identify of outlier loci representing true signals of selection from those resulting from drift. Several of the exposures faced by the population living in the region of the Chernobyl Nuclear Power Plant are known to be mutagenic. As we explore the genomes of the dogs at this location, we aim to identify genome variants that were potentially induced by the prevailing multigenerational exposures and then subjected to ongoing selective pressures to maintain them in the population. Our long-term goal with this unique population of dogs is to establish further evidence to evaluate the degree of local adaptation and thus develop measures of the impact of the exposures experience by these dogs.

## Conclusion

Study of the dogs living within the CEZ offers a unique opportunity to examine the long-term genetic and health consequences of multi-generational exposure to radiation, heavy metals, organic compounds, and other environmental toxicants. The present study takes steps towards understanding these consequences of these adverse environmental exposures by classifying the genetic structure of these populations. Our findings demonstrate genetic population differentiation between the two sampled dog populations at the Nuclear Power Plant and Chernobyl City. Despite having similar breed makeup and being separated by only a short geographic distance, these free-breeding dog populations are reproducing independently of each other and co-occur with little gene flow. Through our outlier analysis, we identified genomic regions that have diverse allele frequencies between the populations, including candidate genes such as *xrcc4* and *cntnap2*. Our findings are likely to inform future studies, where we intend to search these genomic regions and candidate genes for variants, novel and previously documented, to further evaluate the degree of local adaptation within the Nuclear Power Plant and Chernobyl City populations. This approach permits us to pursue the identification of local adaptation as we continue to study the genetics of this distinctive group of dogs. This work, and future studies with these canine populations, will advance our broader understanding on the genetic effects of prolonged exposures to both radiation and non-radiation toxic exposures, and the findings potentially more broadly applicable to the adverse health effects of other environmental nuclear and non-nuclear disasters in both animal and humans.

## Supplementary Information

**Additional file 1.** This file contains supplementary figures 1-3 including a plot of individual inbreeding coefficients, a breed PCA and plot of highest breed match, and a correlation plot for ROH measures compared to breed measures.

**Additional file 2: Supplementary table 2.**

## Data Availability

The datasets used and analyzed during the current study are available from the corresponding author on reasonable request. Publicly available data that was accessed for the current study is available from DRYAD at 10.5061/dryad.078nc [34, 35].

## Abbreviations

CNPP: Chernobyl Nuclear Power Plant
IAEA: International Atomic Energy Agency
CEZ: Chernobyl Exclusion Zone
NPP: Nuclear Power Plant (dog population)
CC: Chernobyl City (dog population)
SNP: Single Nucleotide Polymorphism
MAF: Minor allele frequency
PCA: Principal component analysis
DAPC: Discriminant analysis of principal components
ROH: Runs of homozygosity
Kb: Kilobase
M: Mutation scaled migration rate (migration/mu)
Θ: Mutation adjusted population size (4Ne*mu)
GSD: German shepherd dog
GO: Gene Ontology
ISF: Interim storage facilities
