# Supplementary material for: Population dynamics and genome-wide selection scan for dogs in Chernobyl
Source: Canine Med Genet. 2023 Mar 8;10:1. doi: 10.1186/s40575-023-00124-1 (PMC9993684; doi:10.1186/s40575-023-00124-1)
Supplement: Supplementary file 1 — Additional file 1. This file contains supplementary figures 1-3 including a plot of individual inbreeding coefficients, a breed PCA and plot of highest breed match, and a correlation plot for ROH measures compared to breed measures. [file 40575_2023_124_MOESM1_ESM.pdf]

Supplementary Figures

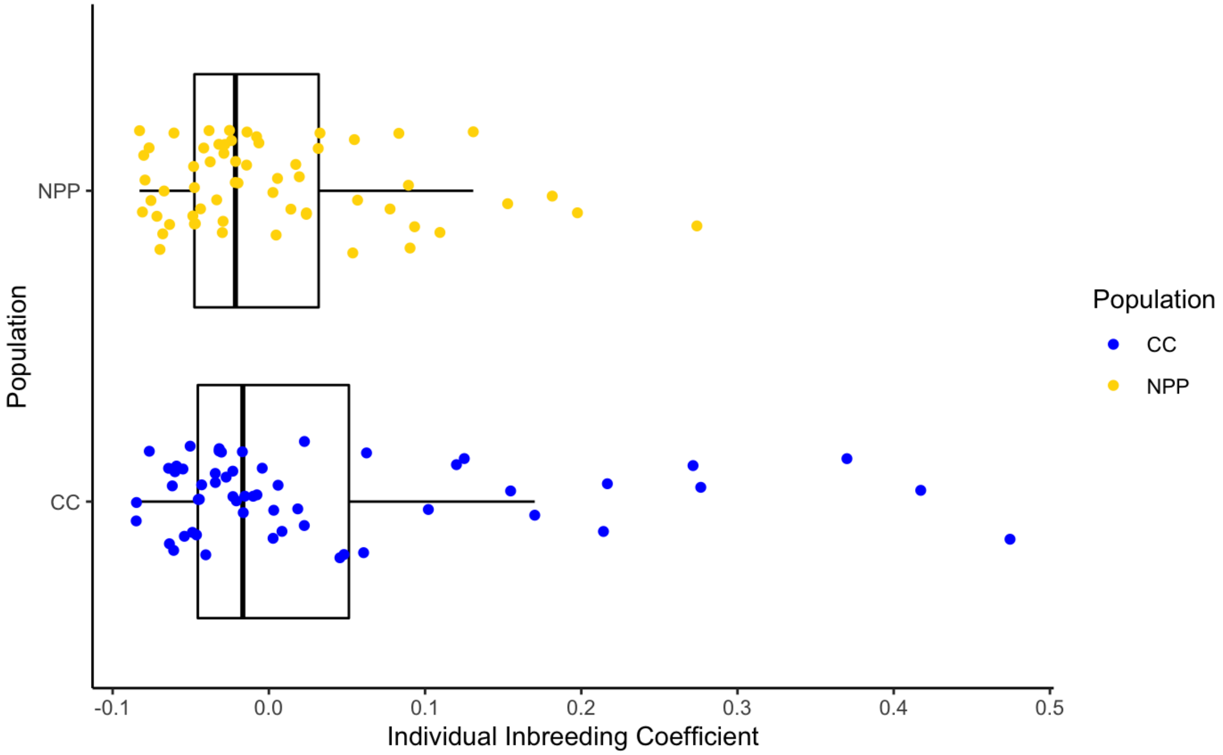

Supplementary Figure 1. Individual inbreeding coefficients plotted based on population. Individual inbreeding coefficients were calculated using the R package SNPRelate. There was no significant difference found between the two populations.

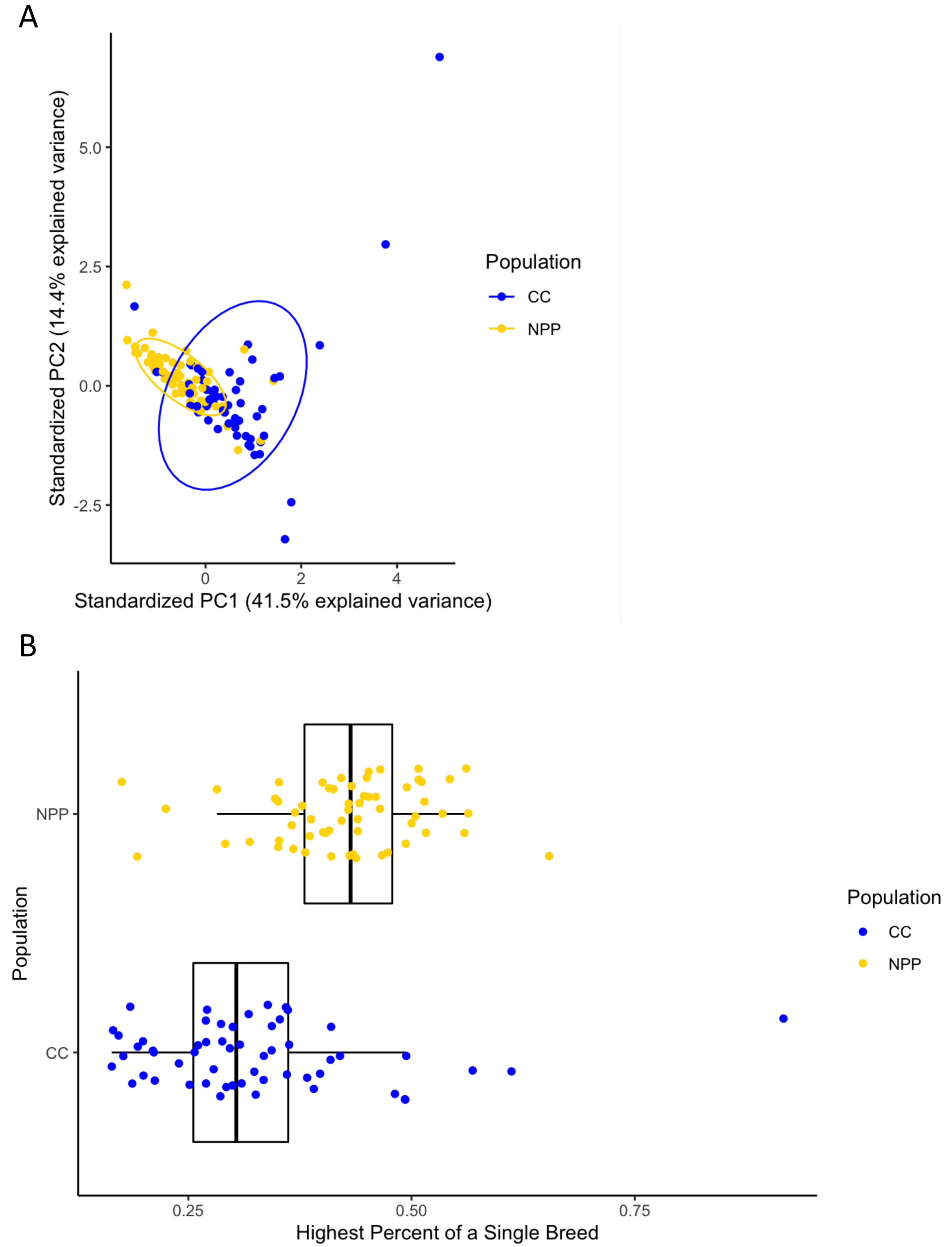

Supplementary Figure 2. Breed measures for each population. A) Plot of PCA based on Wisdom Panel breed match results with little separation based on population. B) Highest percent match of a single breed for an individual, plotted by population.

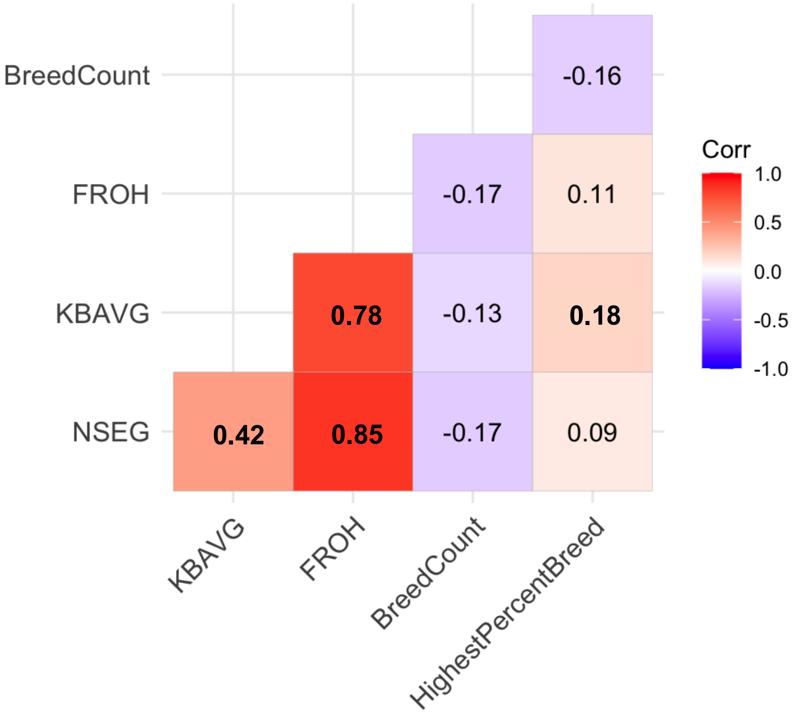

Supplementary Figure 3. Pearson's correlation coefficient heat map for ROH measures compared to breed measures. Bold correlation coefficients indicate significance ($p < 0.05$). KBAVG = average size of ROH, NSEG = Number of ROH segments, FROH = F_{ROH} , BreedCount = number of breed matches in an individual, HighestPercentBreed = maximum breed match percentage in an individual.
